# Dr. Emory C. Abbey

**Published:** 1902-07

**Authors:** 


Dr. Emory C. Abbey, of North Tonawanda, died at the home of his daughter, Mrs. E. C. McDonald, aged 70 years. Dr. Abbey was born in Leicester, in 1832. He was a practising physician for many years, but in the spring of 1886, having given up his professional work, he became the proprietor of the Buffalo Last Works.

